# A rapid and quantitative method to detect human circulating tumor cells in a preclinical animal model

**DOI:** 10.1186/s12885-017-3419-x

**Published:** 2017-06-23

**Authors:** Shih-Hsin Tu, Yi-Chen Hsieh, Li-Chi Huang, Chun-Yu Lin, Kai-Wen Hsu, Wen-Shyang Hsieh, Wei-Ming Chi, Chia-Hwa Lee

**Affiliations:** 1 Department of Surgery, School of Medicine, College of Medicine, Taipei Medical University, Taipei, Taiwan; 2 Division of Breast Surgery, Department of Surgery, Cathay General Hospital, Taipei, Taiwan; 3 Breast Medical Center, Taipei Medical University Hospital, Taipei, Taiwan; 4 Taipei Cancer Center, Taipei Medical University, Taipei, Taiwan; 5 Comprehensive Cancer Center of Taipei Medical University, Taipei, Taiwan; 6 PhD Program for Neural Regenerative Medicine, College of Medical Science and Technology, Taipei Medical University, Taipei, Taiwan; 7 Department of Endocrinology, Cathay General Hospital, Taipei, Taiwan; 8 Institute of Bioinformatics and Systems Biology, National Chiao Tung University, Hsinchu, Taiwan; 9 Research Center for Tumor Medical Science, China Medical University, Taichung, Taiwan; 10 Department of Laboratory Medicine, Shuang Ho Hospital, Taipei Medical University, Taipei, Taiwan; 11 School of Medical Laboratory Science and Biotechnology, College of Medical Science and Technology, Taipei Medical University, Taipei, Taiwan

**Keywords:** Cancer metastasis, Circulating tumor cells, Quantitative PCR, In vivo bioluminescent imaging system

## Abstract

**Background:**

As cancer metastasis is the deadliest aspect of cancer, causing 90% of human deaths, evaluating the molecular mechanisms underlying this process is the major interest to those in the drug development field. Both therapeutic target identification and proof-of-concept experimentation in anti-cancer drug development require appropriate animal models, such as xenograft tumor transplantation in transgenic and knockout mice. In the progression of cancer metastasis, circulating tumor cells (CTCs) are the most critical factor in determining the prognosis of cancer patients. Several studies have demonstrated that measuring CTC-specific markers in a clinical setting (e.g., flow cytometry) can provide a current status of cancer development in patients. However, this useful technique has rarely been applied in the real-time monitoring of CTCs in preclinical animal models.

**Methods:**

In this study, we designed a rapid and reliable detection method by combining a bioluminescent in vivo imaging system (IVIS) and quantitative polymerase chain reaction (QPCR)-based analysis to measure CTCs in animal blood. Using the IVIS Spectrum CT System with 3D–imaging on orthotropic-developed breast-tumor-bearing mice.

**Results:**

In this manuscript, we established a quick and reliable method for measuring CTCs in a preclinical animal mode. The key to this technique is the use of specific human and mouse *GUS* primers on DNA/RNA of mouse peripheral blood under an absolute qPCR system. First, the high sensitivity of cancer cell detection on IVIS was presented by measuring the luciferase carried MDA-MB-231 cells from 5 to 5x10^11^ cell numbers with great correlation (R^2^ = 0.999). Next, the MDA-MB-231 cell numbers injected by tail vein and their IVIS radiance signals were strongly corrected with qPCR-calculated copy numbers (R^2^ > 0.99). Furthermore, by applying an orthotropic implantation animal model, we successfully distinguished xenograft tumor-bearing mice and control mice with a significant difference (*p* < 0.001), whereas IVIS Spectrum-CT 3D–visualization showed that blood of mice with lung metastasis contained more than twice the CTC numbers than ordinary tumor-bearing mice. We demonstrated a positive correlation between lung metastasis status and CTC numbers in peripheral mouse blood.

**Conclusion:**

Collectively, the techniques developed for this study resulted in the integration of CTC assessments into preclinical models both in vivo and ex vivo, which will facilitate translational targeted therapy in clinical practice.

**Electronic supplementary material:**

The online version of this article (doi:10.1186/s12885-017-3419-x) contains supplementary material, which is available to authorized users.

## Background

Cancer metastasis is the process whereby cancer cells spread from the primary tumor to one or more other places in the body. More than 90 % of cancer-associated deaths are directly related to cancer distant metastasis [1]. In fact, all cancers can form metastatic tumors, including cancers of the blood and the lymphatic system (leukemia, multiple myeloma, and lymphoma). Metastatic tumor cells spread through two major highways—lymphatic vessels and blood vessels—to form secondary foci at common sites such as the lungs, liver, brain, and bones [2]. The current understanding of cancer metastasis development is mostly derived from mouse models. It is now known that the formation of a metastasis involves a complex molecular cascade through which cancer cells leave the site of the primary tumor (intravasation), enter the blood and/or lymphatic vessels (circulation), and are disseminated to distant anatomical sites (arrest and extravasation), where they can growth of secondary tumors at the target organ site (colonization) [1]. During this process, a group of enzymes called matrix metalloproteases (MMPs) acts as “molecular scissors,” which are secreted by cancer cells to cut through the proteins that inhibit the movement of migrating cancer cells.

The success rate of metastatic cancer cells forming secondary foci in distant organs is very low, with an estimated rate of 0.01% from primary tumor cells [3]. The reasons for this low success rate may include the following: (1) cancer cells normally live tightly connected to their neighbors and the meshwork of proteins surrounding them, and any detachment from other cells can lead to cancer cell death (anoikis); (2) cancer cells are often quite large compared with other blood cells, and they are easily damaged or get stuck when traveling through the vessels, which leads to cell death; and (3) highly heterogeneous cancer cells may be recognized and destroyed by cells in the immune system. Although some types of metastatic cancer can be cured with current therapies, most cannot. Therefore, the first priority of these therapies is to shrink the cancer or slow its growth to help relieve cancer-related symptoms.

Circulating tumor cells (CTCs) are cancer cells that have been shed from the vasculature of a primary tumor and circulate in the bloodstream [4]. Some of these CTCs acquire the capability to extravasate and colonize secondary sites, spreading tumors to distant vital organs and causing the majority of cancer-related deaths. In recent decades, numerous groups have tried to develop new diagnostic assays to detect CTCs in the peripheral blood of tumor patients. By applying these cutting-edge technologies, anti-metastasis therapies for blocking cancer metastasis in patients is now possible. If metastatic cancer cells can be kept dormant, this will transform cancer into a chronic but manageable disease.

With imminent breakthroughs in the recent study of metastasis, three classes of genes have been distinguished—metastasis initiation genes, metastasis progression genes, and metastasis virulence genes—whose gain or loss of function specifically enables tumor cells to circulate, target, penetrate, and colonize distant organs [5]. Metastasis initiation genes provide an opportunity for primary tumor cells to enter circulation. These genes have cell-motility-, invasion-, and angiogenesis-related abilities that enable tumor cells to target the vasculature in the microenvironment, enter circulation, and be disseminated to distant organs [6, 7]. Metastasis progression genes, which contribute to primary tumorigenesis, fulfill additional functions that are more advantageous to the metastasis site [8]. This process acts as a rate-limiting function in primary tumor growth during metastatic colonization. Metastasis virulence genes provide a selective advantage and aggressiveness to secondary colonization sites [9, 10]. These genes rarely present “poor-prognosis” gene-expression signatures in primary tumors. In addition to these metastasis genes, nearly 30 metastasis suppressors have been identified so far [11]. The first metastasis suppressor, nm23 protein, was identified in the mid-1980s. Other metastasis suppressors are well known due to their important functions in cell and molecular biology, such as the cadherin family (E-cadherin), caspase-8, stress-activated MAPK signaling (p38), and tissue inhibitors of metalloproteinases (TIMPs). These genes are responsible for blocking tumor metastasis in the metastasis initiation state, and in the future, they may provide a way to develop novel therapeutic agents for cancer metastasis by targeting metastasis suppressors.

So far, more than 200 clinical trials have incorporated CTC counts as a biomarker in patients during metastasis screening of various types of solid tumors [12], including breast, gastric, and hepatocellular cancers, using density gradient centrifugation, immunomagnetic separation, side population, cell sorting, and further analysis via flow cytometry, reverse transcription polymerase chain reaction (RT-PCR), gene chips, and quantitative PCR (QPCR) [13–15]. QPCR analysis involves the modification of the PCR principle, which preferentially binds to double-stranded DNA, to measure gene expression. Using TaqMan- or SyBGreen-based QPCR platforms has facilitated the understanding of the clinical relevance of the gene expressions of CTCs in both cancer patients and healthy subjects [16, 17]. For instance, higher cytokeratin-7 (CK7) and epidermal growth factor receptor (EGFR) expressions (4- to 8-fold) in cancer cells have been found in lung and breast cancer patients, whereas normal leukocytes are present in a very low level of expressions [18]. Thus, quantification of these metastatic-expressing messenger RNAs (mRNAs) is essential in distinguishing normal expression in blood from that with the presence of CTCs.

In 2005, de Kok and colleagues used GUS genes to normalize the variability between clinical tissue samples using QPCR measurements [19]. In that study, 13 housekeeping gene expressions were measured among 80 epithelial tissue samples, including normal tissue and tissue from colorectal, breast, prostate, skin, and bladder tumors, with different cancer staging, from noninvasive to metastatic carcinomas. The results demonstrated that the expression patterns of hypoxanthine-guanine phosphoribosyl-transferase (HPRT) and the GUS genes were the two most accurate in reflecting the mean expression pattern compared with the other 13 selected genes. The QPCR results showed a very precise accuracy, with ±1.3 and ±1.4 PCR cycles (Ct) in HPRT and GUS normalization, respectively, which indicates that the bias from all clinical tissue samples was less than two times the standard deviation (2-SD).

In this study, we aimed to design a quick and reliable method to monitor human CTCs in a mouse model using both bioluminescent imaging in vivo and QPCR-based analysis. Well-designed primer sets of ß-glucuronidase (GUS) genes for both human and mouse sensitivity were used to detect CTC numbers during cancer metastasis development in orthotropic-developed breast-tumor-bearing mice. In addition, we used the IVIS Spectrum CT System with 3D–imaging (hereafter, IVIS) to clearly illustrate the strong correlation between lung metastasis development and CTC numbers in peripheral mouse blood. The results from this study revealed the molecular basis of CTCs in cancer development, which can be applied as biomarkers to accelerate translational medicine in clinical investigations.

## Methods

### Cell culture

Human mammary gland epithelial adenocarcinoma cell lines MDA-MB-231 was purchased from the American Tissue Culture Collection (ATCC, Manassas, VA) and maintains in DMEM/F12 medium. The cells were incubated with 10% (*v*/v) foetal bovine serum (FBS, Biological Industries, Israel), 100 units/ml penicillin and 100 mg/ml streptomycin in a 37 °C incubator with 5.0% CO_2_.

### Transfection and cell line selection

MDA-MB-231 cells were transfected with pcDNA3 plasmids expressing the firefly luciferase gene (the gene sequences were originally from *luc4.1*; Chris Contag, Stanford University, Stanford, CA, USA) by electroporation, as described previously [20]. Briefly, 5 × 10^6^ cells were washed twice with PBS and mixed with 10 μg of plasmid. Two pulses were applied for 20 milliseconds under 1.2 kV on the pipette-type MicroPorator MP-100 (Digital Bio, Seoul, Korea). The stable cells were selected 48 h later with G418 (6 mg/mL). The bioluminescent derivatives of MDA-MB- 231 cells were used for further in vivo studies.

### Animal experiments

Four-week-old severe combined immunodeficient (SCID) female mice were purchased from the National Science Council Animal Center (Taipei, Taiwan) and housed in micro-isolator cages at the Laboratory Animal Center in National Defense Medical Center (Taipei, Taiwan). This study was carried out in strict accordance with the recommendations in the Guide for the Care and Use of Laboratory Animals from the National Institutes of Health. The protocol was approved by the Institutional Animal Care and Use Committee (IACUC) at National Defense Medical Center (Permit Number: IACUC-15-240). All surgeries were performed under isoflurane anesthesia and all efforts were made to minimize suffering. During the experiment, no stress or abnormal behaviors due to tumor bearing were observed in the mice. The health status of the animals was monitored once daily by a qualified veterinarian. Food and water were replaced every two days.

### Bioluminescent (IVIS) and tumor multimodality (CT/DLIT/FLIT) imaging

Bioluminescent imaging was performed with a highly sensitive, cooled CCD camera mounted in a light-tight specimen box (In Vivo Imaging System - IVIS; Xenogen). For in vivo imaging, animals were given a serial numbers of luciferase stable expressed MDA-MB 231 breast cancer cells (1 X 10^5^, 10^4^, 10^3^, 10^2^ cells and control-PBS only) by tail vein injection. After 15 min, the mice were i.p. injected with D-luciferin (200 mg/kg) for fifteen minutes. Animals were placed onto the warmed stage inside the camera box and received continuous exposure to 2.5% isoflurane to sustain sedation during imaging. Every group of mice was imaged for 30 s. The light emitted from the mice were detected by the IVIS camera system, integrated, digitized, and displayed. Regions of interest from displayed images were identified and were quantified as total photon counts or photons/s using Living Image® software 4.0 (Caliper, Alameda, CA.).

### Orthotropic breast metastasis animal model

The orthotropic tumor model was used to mimic the cancer in humans through use of immune competent and severe combined immune deficiency (SCID) mice (6–8 week old). Five mice were anesthetized with 2% isoflurane and each implanted with 5 × 10^6^ luciferase expressing MDA-MB-231cells into the mammary fat pad. Five mice injected PBS were presented as control. All the mice were scarified for blood collection after 10-week of cancer cell injection. Throughout the study, all mice were kept in an environmentally controlled room with temperature and relative humidity maintained between 69 and 75 F (21–24 C) and 43–65%, respectively.

### Blood samples

100–150 ul of blood was obtained by cardiac puncture from mouse and processed according to standard separation protocols. Total DNA was isolated from human cell lines and mouse leukocyte using AxyPrep blood genomic DNA miniprep kit by following the manufacturer protocol. NanoDrop quantification were used for DNA quantity (260/280) measurement. All DNA samples contained at least 10 ng/ul DNA.

### Real-time quantitative PCR

Human GUS primers (forward: AGTGTTCCCTGCTAGAATAGATG and reverse: AAACAGCCTGTTTACTTGAG) and mouse GUS primers (forward: GCAGGCTTTCAAGAGTTCA and reverse: TATGAGCTGGTCCTCCATTTC) were synthesised by Genomics BioSci and Tech (Taipei, Taiwan). A LightCycler thermocycler (Roche Molecular Biochemicals, Mannheim, Germany) was used for QPCR analysis. One microliter of sample and master-mix were first denatured for 10 min at 95 °C and then incubated during 40 cycles: denaturation at 95 °C for 5 s; annealing at 60 °C for 5 s; elongation at 72 °C for 10 s and detected for fluorescent intensity. The PCR samples were all performed melting curve analysis for non-specific PCR product detection. The human GUS fluorescence intensity was measured and normalised to the mouse GUS expression by using the built-in Roche LightCycler Software, Version 4.

### Absolute quantitative QPCR

For generate the absolute quantitative standard curve for QPCR analysis. We used PCR product of mouse GUS gene and cloned into TA cloning vector (*pTA*® Easy Cloning Kit) which purchased from Genomics BioSci and Tech (Taipei, Taiwan). After following the steps of gene sequence, E.coli amplification, plasmid purification and determination of molecular weight, the copy number of GUS gene were calculated and diluted into 10^8^ to 10^2^ per μl. Each copy number of GUS gene were measured with its accuracy and the liner correlation.

### Statistical methods

All data were expressed as mean ± SD and performed student *t*-test analysis for the pairwise samples. All statistical comparisons were performed using the SigmaPlot graphing software (San Jose, CA, USA) and the Statistical Package for the Social Sciences v.13 (SPSS, Chicago, IL, USA). A *P*-value <0.05 was considered statistically significant and all statistical tests were two-sided.

## Results

### Sensitivity of cell numbers detected by the IVIS

**Fig. 1:**
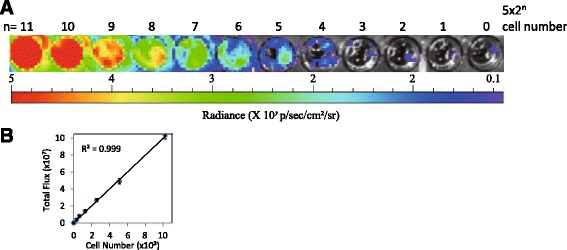
In vitro bioluminescence calculation of MDA-MB-231. **a** Luciferase-expressing MDA-MB-231 cells were serially diluted in wells from 10,240 to 5 cells/well. Luciferin was added to each well and the plate was imaged with radiance flux (photons/s/cm^2^/sr). The range of bioluminescence was collected from three experiments. **b** Total radiance flux from each well was compared to their cell number per well. The correlation between mean radiance flux and injected cell numbers are indicated as R^2^ values. The error bars represent three independent assays

An IVIS imaging system is a great device for observing fluorescent, chemo, and biosensor lights in vitro and in vivo. In order to monitor the cancer cells in the xenograft tumor mice detected by the IVIS, we established MDA-MB-231 luciferase-expressing breast cancer cells for this study. Briefly, a luciferase-containing vector was introduced into the MDA-MB-231 breast cancer cells, selected by G418 (geneticin), for one month. Colonies of MDA-MB-231 luciferase-expressing breast cancer cells were chosen and expanded for further study. Figure 1a demonstrates that the IVIS was highly sensitive and accurate regarding the photon measurement of serial diluted breast cancer cell numbers. The photons of the luciferase gradient lights ranged from high radiance (red color) to low radiance (dark-blue color). As shown in Fig. 1a, the wells containing 5 × 2^9^ to 5 × 2^11^ cells were detected and presented in red, whereas the wells containing 5 × 2^0^ to 5 × 2^3^ cells showed no significant color change.

In Fig. 1b, we correlated the radiance influx from each well with the breast cancer cell number input. The results of the correlation demonstrated a straight standard curve, indicating the reliability of IVIS detection in cell measurement, and the R-squared (R^2^) value was 0.999. The data also illustrated that the high sensitivity of IVIS detection, even in a small number of cells (e.g., five cells), could still be measured in vitro, ensuring the reliability of the CTC numbers in animals.

### CTC detection using the IVIS

An adult mouse general weighs around 20 g, of which one-thirteenth is blood (1.54 ml). In a healthy physical condition, the white blood cell (WBC) count normally accounts for 6 to 15 X 10^3^ per mm^3^. That means an adult mouse has around 1.5 X 10^7^ WBCs circulating in its blood, which makes it difficult to detect a small population of circulating tumor cells. In this study, we used tail-vein injections to transfer 10^2^, 10^3^, 10^4^, and 10^5^ MDA-MB-231 breast cancer cells (carrying luciferase-expressing genes) and a control solution (phosphate-buffered saline, PBS) into the mice. After 15 min, the mice were given an intraperitoneal (IP) injection of D-luciferin (200 mg/kg), and then the photon flux of the dorsal and ventral views of the mice were measured by the IVIS, respectively.

**Fig. 2:**
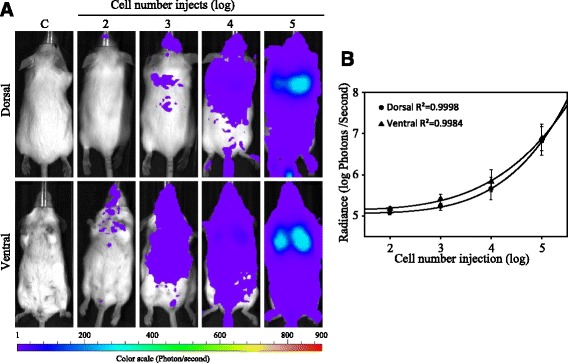
In vivo bioluminescence measurement of MDA-MB-231 cells in the animal model. **a** 10^2^ to 10^5^ of luciferase-expressing MDA-MB-231 cells were injected into mice. Mice with PBS (no cells) were included as controls. Luciferin substrate was IP injected into each mouse and imaged to obtain radiance flux (photons/s), with dorsal and ventral positions. The data of bioluminescence for each mouse was collected from three experiments. **b** Total radiance flux from each mouse was compared with the known breast cell numbers. The correlation of dorsal and ventral views between the mean radiance flux and the injected cell numbers are indicated as R^2^ values. The error bars represent three independent assays

As can be seen in Fig. 2a, strong photon signals were detected in the mice that were injected with 1 X 10^5^, 10^4^, and 10^3^ breast cancer cells in the ventral view, whereas strong photon signals were detected in the mice injected with 1 X 10^5^ and 10^4^ breast cancer cells in the dorsal view. Due to the tail-vein injections for blood circulation, lung tissue was the first organ that the breast cancer cells entered, where they were trapped by pulmonary capillary vessels. As a result, a higher luciferase signal was found in the lung tissue, whereas in other parts of the mice, homogeneous luciferase signals were distributed. For the control mice, no photon signals were detected in either the ventral view or the dorsal view.

In Fig. 2b, we correlated the cell number injections with the luciferase signals from the IVIS measurements, and the data showed that the photon flux in both the dorsal and ventral views of the mice exactly reflected the number of cells injected, and the R^2^ values were 0.9998 and 0.9984, respectively. This data clearly illustrates that by using stable luciferase-expressing cells in the xenograft models, even a very small number of cancer cells could be detected by the IVIS, with a limitation of approximately 100 CTCs in the mice’s bloodstream.

### Generating absolute QPCR analysis

**Fig. 3:**
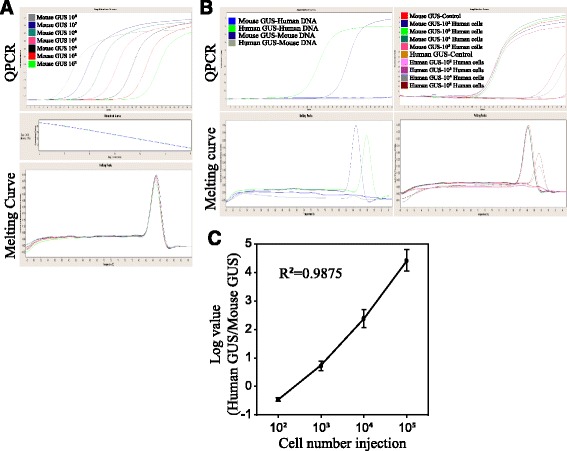
QPCR evaluation of human CTC numbers in peripheral mouse blood. **a** For the generation of absolute QPCR analysis, the mouse GUS PCR products were cloned into a TA vector, followed by gene sequencing, *E. coli* amplification, and plasmid purification. The molecular weight of the plasmid was calculated using the value of OD.260 and diluted into 10^8^ to 10^2^ copy number/μl. **b** For the specificity of GUS gene primers for human- and mouse-purified DNA (upper-left panel), the PCR products were evaluated by melting curve analysis after quantitative analysis for single product confirmation (lower-left panel). DNA from the mouse blood containing human CTCs was measured by applying both human and mouse GUS primer sets. DNA from the mice with PBS (no cells) was included as a control (upper-right panel). The PCR products from the mouse blood containing CTCs were evaluated by melting curve analysis after quantitative analysis for single product confirmation (lower-right panel). **c** The QPCR-calculated cell numbers for each mouse were compared with the known IV-injected breast cell numbers. The human GUS cell numbers were normalized with the corresponding mouse GUS results. The correlation between the human GUS copy numbers and the injected cell numbers are indicated as R^2^ values. Bar errors are represented by three independent experiments

In order to quantify the small number of CTCs in the mice’s peripheral blood, we designed an absolute QPCR system using mouse GUS genes containing plasmid for standard curve generation. The purified plasmids were calculated into 10^8^ to 10^2^ copy number/μl by measuring the molecular weight of OD.260. In Fig. 3a, the linear standard curve of absolute quantification is R^2^ of 1.896, with ample efficiency and 0.0131 in errors. A melting curve indicates that all the PCR products contained the same base pair products without noise band interference. By applying this absolute quantification system, we were able to count the CTCs during cancer metastasis development.

### Establishment of real-time PCR used in human- and mouse-specific DNA primer sets

In this study, in order to detect the number of human breast cancer cells circulating in the mice’s blood, we designed paired GUS QPCR primers specifically for human and mouse genes. Concerning the specificity of human GUS primers, we tested six paired primers targeting human GUS sequences. In Additional file 1: Figure S1, the QPCR data shows that the human GUS primers obtained the best specificity in defining human and mouse DNA, whereas the other primers accidentally detected fluorescence signals at 30 to 37 cycles using human GUS1 to GUS5 primers for QPCR detection. Next, we wanted to know whether or not both human- and mouse-specific primers would detect the target genes without a cross-reaction. Additional file 2: Figure S2 shows that the sequences of human GUS primers were not identical to the homolog site of the mouse GUS gene, indicating a high specificity of human GUS primers. In Fig. 3b (upper-left panel), QPCR specifically detected human (green curve) and mouse (dark-blue curve) DNA by adding their specific GUS primers to the mouse/human DNA mixture, respectively. In contrast, human GUS primers could not detect mouse DNA (gray curve) and mouse GUS primers could not detect human DNA (blue curve) in a 40-cycle QPCR analysis. Moreover, Fig. 3b (bottom-left panel) shows that the dissociation temperatures for the human and mouse GUS PCR products were 88.5 °C and 84 °C, respectively. The melting curve analysis also demonstrates the high specificity of the GUS primer sets designed for humans and mice.

### CTC detection using QPCR analysis

Next, we investigated whether or not QPCR analysis could be applied to CTC measurement in animals. The mice in Fig. 2a had blood drawn from their heart tissue, which was collected in a tube containing ethylenediamine tetra-acetic acid (EDTA). The blood was then added to a red blood cell (RBC) lysis buffer to remove the RBCs, and then centrifuged for WBC enrichment. Next, the cells were subjected to DNA purification and QPCR using both human and mouse GUS-specific primers, respectively. As shown in Fig. 3b (upper-right panel), the blood from each mouse contained a similar number of cells (mouse GUS primers), which detected fluorescence signals at around 20 PCR cycles. Based on the small amount of human cancer cells in the mice’s blood, we detected human DNA signals at 32 cycles at 10^5^, 35 cycles at 10^4^, and 39 cycles at 10^3^ MDA-MB-231 in the injected mice. The melting curve of all the PCR products illustrates the specificities of both human and mouse primers in detecting human metastatic cells in peripheral mouse blood. Next, we correlated the QPCR results with the breast cancer cell injections. Figure 3c demonstrates a consistent correlation between the cell number injections and the QPCR analysis, with a reliable linear curve R^2^ value of 0.9875.

### CTC detection in an orthotropic mouse model

The transgenic tumor model and subcutaneously-growing human tumors in immune-deficient mice are the most frequently used rodent tumor models. However, the limitation of these models is that they do not represent clinical cancer development, especially with regard to metastasis and drug sensitivity. MDA-MB-231 breast cancer belongs the triple-negative breast cancer (TNBC), which represents strong cancer metastasis and is a common cell line in animal models. For these reasons, we used orthotropic implantation to transplant histologically-intact fragments of MDA-MB-231 human breast cancer cells into the corresponding organ in immune-deficient rodents.

**Fig. 4:**
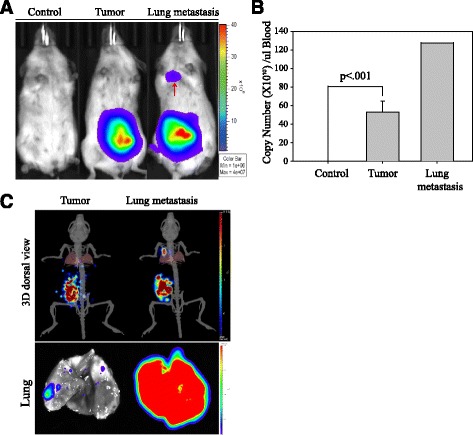
In vivo bioluminescence of MDA-MB-231 in the orthotropic animal model. **a** 5 X 10^6^ luciferase-expressed MDA-MB-231 cells were IP injected orthotropically into the mammary fat pad. The mice with PBS were the control group. All mice were fed a normal diet for two months. For IVIS imaging, luciferin substrate was IP injected into each mouse and imaged to obtain radiance flux (photons/s). The red arrow indicates a lung metastasis signal. **b** For the QPCR calculation of CTC cell numbers from the control and breast-tumor-bearing mice, the human GUS cell numbers were normalized with the corresponding mouse GUS results. Bar errors are represented by three independent experiments, and the *p* value represents the significance between the control and the tumor-bearing mice. **c** The upper-panel shows the IVIS Spectrum CT imaging of the subcutaneous tumor model for both breast-tumor-bearing and lung metastasis mice. The mice were positioned in a dorsal view. The lower-panel shows the IVIS images of lung tissues

As can be seen in Fig. 4a, two months after the orthotropic xenograft tumor implantation, one of the five breast-tumor-bearing mice developed a significant lung metastasis, with strong luciferase activity in its lung tissue (red arrow). The IVIS image also shows that the breast-tumor-bearing mice had extremely high luciferase activity, compared with the PBS-injected control group. We next applied human and mouse GUS QPCR detection to the mice with/without orthotropic xenograft tumors, as shown in Fig. 4b. The absolute QPCR analysis clearly shows that the mice with the xenograft tumors obtained a significantly higher copy number of CTCs, compared with the control mice (53.6 ± 12 v.s. 0 copy number/200 μl blood, *P* < .001), whereas the mouse with the lung metastasis measured 128 copy number/200 μl in peripheral blood. In order to define the tumor and the distance of the metastasis locations in the mice, we used the IVIS for the gravity model (see Fig. 4c, upper panel).

Additional file 3: Movie S1 clearly demonstrates the bioluminescence-detected signals from the surface orthotropic tumor region, whereas Additional file 4: Movie S2 shows strong bioluminescence signals deep inside the lung tissue. Finally, the mice were scarified to remove lung tissue and confirm breast cancer metastasis (see Fig. 4c, lower panel). The lung tissue with cancer metastasis showed high homogeneous luciferase activity, whereas the other lung tissues showed weak luciferase activity. This data clearly shows that the highly sensitive and specific QPCR detection accurately reflected cancer metastasis development, even with a very small number of CTCs in blood circulation.

## Discussion

QPCR has been widely used for the detection of CTCs in the peripheral blood of various types of cancers [21–25]. However, the sensitivity and specificity of CTC detection can be extremely variable based on the experimental design, the markers chosen, and the method of cell enrichment. Among these factors, a reliable and detectable CTC marker is one of the most important in determining whether or not the experiment will be a success. To date, reference control genes are the most frequently used method of normalizing the mRNA concentration in samples. These reference control genes are often referred to as “housekeeping genes,” which remain unchanged in the tissues or cells under investigation. In our experimental design, the GUS gene was selected to identify the cells of humans and mice. GUS degrades glycosaminoglycans, including heparan sulfate, dermatan sulfate, and chondroitin-4,6-sulfate. In molecular biology, the GUS reporter gene system is a powerful tool that is often used for the assessment of gene activity in mammalian and plant cells. Therefore, monitoring β-glucuronidase activity through the use of a GUS assay can determine the spatial and temporal expression of the gene in question [26]. GUS genes are essential for cell biology and constitutive action, and an increasing number of studies have used them for mRNA normalization.

The results from the two experimental mRNA and DNA detections in the target genes using QPCR analysis showed varying levels of CTCs in the blood samples, and each had advantages and limitations according to their expression and stability. Taking mRNA as an example, due to its instability, the reverse transcription process was required to transform mRNA into complementary DNA (cDNA) under PCR experimentation, specifically, the denaturation step of PCR. When a constitutive-expression housekeeping gene was selected, the cDNA copy number included an abundance of target genes. However, trying to detect a high similarity of genes in two samples (e.g., the human and mouse GUS genes in this study) was a difficult task when designing primers. By comparison, the gene sequences of GUS in the human and mouse mRNA in the coding regions showed 77.8% of shared identity, which resulted in difficulty in finding primer sets that would precisely measure one gene expression without disturbing the other. On the other hand, high stability is one of the best-known advantages of using DNA as a template because CTCs can be measured using purified DNA or enriched leukocyte during QPCR analysis.

The long sequence of DNA (including exon and intron) also provides more possibilities and easier conditions for designing suitable PCR primers for target genes in different species. Except for gene duplication (also referred to as gene amplification), a significant drawback of using DNA as a template for cell quantification is its lower sensitivity during QPCR measurement, compared with gene expression detection. This results in researchers using more blood or leukocyte taken from the mouse, or adding more amplification cycles during PCR analysis to overcome this natural defect. Thus, using mRNA reverse transcription cDNA as the source template and having well-designed target primer sets is a better strategy when evaluating the cell numbers of human CTCs in peripheral mouse blood using QPCR analysis.

Human xenograft tumors implanted in immunocompromised mice provide an important approach for the assessment of tumor growth, invasion, metastasis, angiogenesis, and the effects of the tumor’s microenvironment [27]. However, it is difficult to investigate CTCs numbers in xenograft tumor models during cancer development. In recent decades, using bioluminescence and fluorescence has made it easier to address these issues, especially in xenograft models. Moreover, using an IVIS provides a method that facilitates and enhances the quantification of tumor progression and treatment efficacy by measuring the progression in the bioluminescence or fluorescence associated with tumor growth. In this study, we found that the luciferase measurement from the IVIS showed a linear and trustworthy correlation with the number of CTCs. In addition, the metastatic cancer that developed in the secondary tissue infiltration was also detected by the IVIS through its 3D–images of blood-enriched organs.

Finally, using QPCR-based primer sets to quantify the cell numbers of the CTCs in the xenograft mouse model with human breast cancer cells, we compared both bioluminescent imaging and QPCR measurements in the mice’s bloodstream to examine the replicability and reliability of the whole system. The data from the orthotropic breast tumor animal model illustrated the most direct and trustworthy evidence in this preclinical study, which showed a positive correlation between the metastasis event and the CTC numbers. This technique lays a strong foundation for future studies to examine therapeutic responses and answer biological questions involving CTC-associated molecules and signaling pathways during cancer metastasis. However, the limitation for this study is that only one type of human breast cancer cell was tested in animals. Ideally, CTC detection using realtime PCR in preclinical animal should involve different types of cancer cells to ensure human GUS expression can be precisely measured in mouse blood. Furthermore, the amount of blood collected from mice could be little, from volume 50–500 ul in submandibular blood collection or tail-vein blood collection. This would cause less sensitivity of CTC detection in animal model. In clinic, to overcome this limitation, all CTC related clinical trials have used cell enrichment as an essential and necessary step to increase the sensitivity of CTC detection.

The results from the current investigation should facilitate the discovery of novel therapeutic targets and the development of specific inhibitors and drugs for clinical practice. We expect that this preclinical system involving the IVIS and QPCR analysis will accelerate the biological profiling of CTCs, which will largely improve the diagnostic capabilities used in clinical oncology. In this study, we used breast cancer as a target for CTCs; however, this technique should also be applicable to mouse models for other human cancers, with important implications for cancer metastasis therapy.

## Conclusions

In conclusion, by combining IVIS and QPCR-based analysis, we are able to quantitative CTC numbers in mouse peripheral blood to understand the tumor progression in mammary xenograft carcinoma model. In addition, the information from CT system with 3D–imaging dramatically improves the identification of earlier metastatic tumors.

## Additional files

Additional file 1: Figure S1. Designing suitable human GUS primers for a xenograft animal model. (A)-(F) panels used primers designed by Roche LightCycler Probe Design Program targeting human GUS genes. Both human and mouse DNA were used to measure the primers’ specificity. (PDF 525 kb)

Additional file 2: Figure S2. The similarity of human and mouse GUS primer sequences. Human and mouse GUS sequence homolog analysis was performed by multiple sequence alignment of DNA STAR. The red-framed square indicates the forward and reverse sequences of the human GUS primer, and the discontinued red and blue bars represent identical and non-identical sequences, respectively. (PDF 320 kb)

Additional file 3: Movie S1. 3D image of in vivo bioluminescence in a non-invasive orthotropic animal. (AVI 615 kb)

Additional file 4: Movie S2. 3D image of in vivo bioluminescence in a lung metastasis animal model. (AVI 533 kb)

## Abbreviations

CTCs: Circulating tumor cells
GUS: ß-glucuronidase
I.P.: Intraperitoneal
IVIS: In vivo imaging system
QPCR: Quantitative PCR
SCID: Severe combined immune deficiency
TNBC: Triple-negative breast cancer
