# Supplementary figures and images for: A rapid and quantitative method to detect human circulating tumor cells in a preclinical animal model
Source: BMC Cancer. 2017 Jun 23;17:440. doi: 10.1186/s12885-017-3419-x (PMC5481956; doi:10.1186/s12885-017-3419-x)

Supplementary figure 1

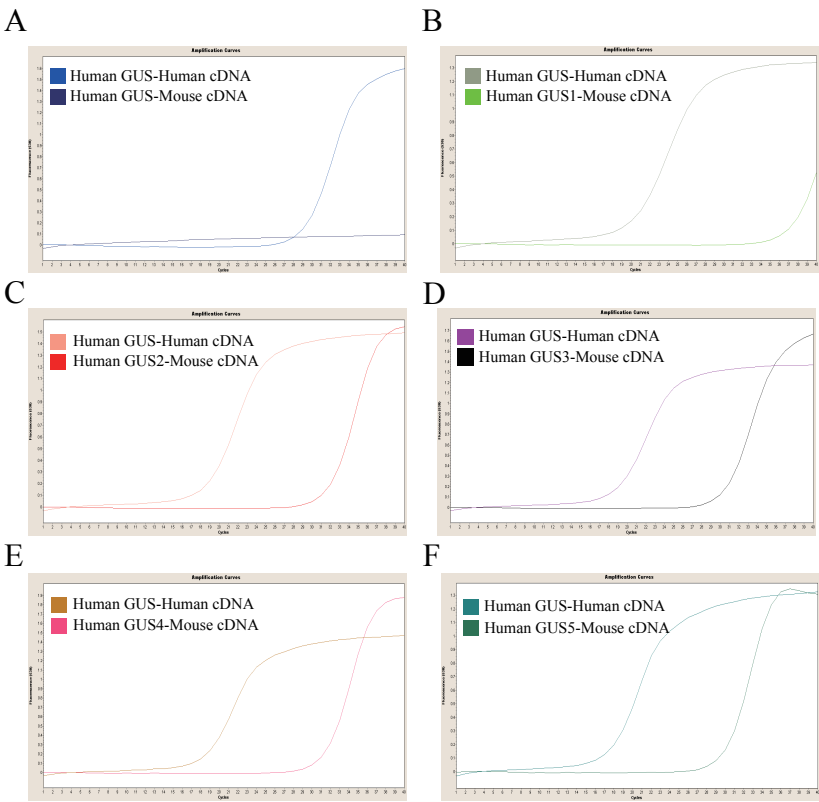

Supplement: Supplementary file 1 — Designing suitable human GUS primers for a xenograft animal model. (A)-(F) panels used primers designed by Roche LightCycler Probe Design Program targeting human GUS genes. Both human and mouse DNA were used to measure the primers’ specificity. (PDF 525 kb) [file 12885_2017_3419_MOESM1_ESM.pdf]

## Supplementary figure 2

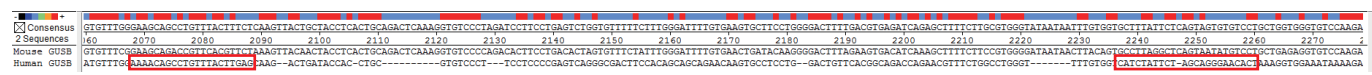

Supplement: Supplementary file 2 — The similarity of human and mouse GUS primer sequences. Human and mouse GUS sequence homolog analysis was performed by multiple sequence alignment of DNA STAR. The red-framed square indicates the forward and reverse sequences of the human GUS primer, and the discontinued red and blue bars represent identical and non-identical sequences, respectively. (PDF 320 kb) [file 12885_2017_3419_MOESM2_ESM.pdf]
